# Rationale for the Use of CAD/CAM Technology in Implant Prosthodontics

**DOI:** 10.1155/2013/768121

**Published:** 2013-04-16

**Authors:** Jaafar Abduo, Karl Lyons

**Affiliations:** ^1^School of Dentistry, Melbourne University, 720 Swanston Street, Carlton Melbourne, VIC 3010, Australia; ^2^Department of Oral Rehabilitation, Faculty of Dentistry, University of Otago, 310 Great King Street, Dunedin 9054, New Zealand

## Abstract

Despite the predictable longevity of implant prosthesis, there is an ongoing interest to continue to improve implant prosthodontic treatment and outcomes. One of the developments is the application of computer-aided design and computer-aided manufacturing (CAD/CAM) to produce implant abutments and frameworks from metal or ceramic materials. The aim of this narrative review is to critically evaluate the rationale of CAD/CAM utilization for implant prosthodontics. To date, CAD/CAM allows simplified production of precise and durable implant components. The precision of fit has been proven in several laboratory experiments and has been attributed to the design of implants. Milling also facilitates component fabrication from durable and aesthetic materials. With further development, it is expected that the CAD/CAM protocol will be further simplified. Although compelling clinical evidence supporting the superiority of CAD/CAM implant restorations is still lacking, it is envisioned that CAD/CAM may become the main stream for implant component fabrication.

## 1. Introduction

For over three decades, evidence to support the validity of oral implants as a treatment option to replace missing teeth has been accumulating. The impressive performance of oral implants has motivated manufacturers and researchers to propose more innovative and convenient treatment protocols. Simpler protocols have allowed a greater number of clinicians to provide implant treatment for a wider range of patients while maintaining a predictable treatment outcome. More recently, one of the major developments in implant prosthodontics has been the adoption of engineering principles in the form of computer-aided design and computer-aided manufacturing (CAD/CAM) to construct implant prosthesis. By reverse engineering the oral implant, it was envisioned that the prosthetic components could be designed and manufactured to a similar quality and predictability to industrial workpieces [1–3].

In industry, the benefits of computerized engineering technology include high precision, simpler fabrication protocol and minimal human intervention. These advantages make CAD/CAM ideal for quality assurance, precision production and cost effective manufacturing [3]. Because of this, it is no surprise that the CAD/CAM technology has been adopted in dentistry [1, 4]. Today, CAD/CAM is the only means of producing durable tooth-colored and metal-free components in dental practice, including implant dentistry, and also provides the option of chair-side fabrication of indirect restorations. The aim of this narrative review is to critically evaluate the current knowledge regarding the rationale of CAD/CAM implant abutments and frameworks.

## 2. Requirements for Implant Abutments and Frameworks

Implant prosthetic components should exhibit sufficient durability to withstand functional loading without distortion or fracture. In addition to functional loads, the prosthetic components are subjected to an excessive amount of preload stress following torquing the retaining screws. Therefore, implant component material should also be selected according to their ability to resist fracture in thin section. This criteria is more critical to ceramic abutments which are more brittle and susceptible to fracture in thin sections [5, 6].

Correct external contour of implant abutments and frameworks will provide clearance for the restorative material which is needed to attain ideal aesthetics and durability of the definitive restoration [7]. Employment of an anatomical contour has been found to minimize the risk of veneering ceramic chipping [8–11]. Further, implant prosthetic components should exhibit a natural emergence profile that mimics natural tooth contour to support the peri-implant soft tissues [12–14]. Suitable soft tissue support will also facilitate successful aesthetic integration of the prosthesis.

In the case of the cement-retained restoration, ideal abutment geometry is required to provide resistance and retention form for the definitive dental prosthesis [15, 16]. To facilitate cementation material removal, the finish line should be closely related to the soft tissue contour and follow the mucosal outline [12, 13].

The prosthetic components should exhibit accurate fit on the implant, which implies simultaneous and even contact of all the fitting surfaces [17]. It has been proposed that an accurate fit of the implant components will minimize bacterial leakage and the strains within the implant components and the peri-implant bone. Subsequently, the biological and mechanical complications, such as bone loss and components loosening or fracture, will be reduced [17].

In order to endorse the long term performance of an implant restoration, the components should be biocompatible. Since the most commonly applied materials are noble metals, commercially pure titanium, titanium alloys, and ceramics (alumina and zirconia), the vast majority of patients with oral implants are served with biocompatible restorations [13]. The typical aesthetic limitation of metallic oral implant components is the greyish coloration of the mucosal tissues, especially for thin gingival biotype situations. Several authors have reported the advantage of using ceramic abutments to overcome the undesirable gingival discoloration [18, 19].

## 3. Traditional Methods for Constructing Implant Prostheses

Two traditional approaches are available for implant abutment and framework construction, namely, stock abutments and the lost wax/casting approach. Stock abutments are provided by all the implant suppliers and are milled in a similar way to an implant fixture. The available materials are commercially pure titanium, titanium alloys, and zirconia. Since stock abutments are industrially produced in well-controlled conditions, they exhibit superior durability and fit accuracy than cast abutments [20]. The fit of stock abutments was evaluated and found to have a vertical gap of 5.6 *μ*m which was about half the vertical gap of cast abutments [21]. However, customization is limited to grinding the external surface to provide clearance for the restorative material. Although this might be acceptable for titanium abutments, it has been shown to reduce the overall strength of zirconia abutments [6, 22]. Likewise, the finish line is located according to average values which might not necessarily coincide with the existing mucosal contour [13, 14]. Most of the stock abutments are available in cylindrical form which leaves the emergence profile modifiable only by the final crown. Subsequently, to obtain an aesthetic outcome, the margins should be deeply placed which hinders efficient cement removal. Therefore, their use should be restricted to mucosal tissues with minimal scalloping and in less aesthetically demanding situations. 

To overcome the customization limitations of stock abutments, cast abutments have been advocated [23, 24]. In the dental laboratory, the abutment or the framework is fully contoured by wax or resin and conventionally cast. To enhance the fit of the components, cast-on systems have been developed where the fitting surface is not involved in casting. However, although the casting facilitates the customization process, it is labour intensive, and a high level of quality control is mandatory. The numerous steps involved and significant temperature fluctuations have been suggested as the cause of compromised final fit [25, 26]. Misfit is even further accentuated in the framework, where increasing the span of the framework increases the amount of distortion [27]. Because of this, several authors have recommended the incorporation of additional fit modifying techniques such as sectioning and soldering, laser welding, or spark erosion [28]. In addition, due to the continuously increasing cost of noble metals, the cost-efficiency of casting is questionable. Because commercial dental laboratories cannot produce implant components from high-strength ceramics, this technique is only able to provide metallic components.

## 4. CAD/CAM Protocol

The CAD/CAM protocol was initially introduced for tooth-supported restorations for the purpose of simplicity, convenience, and elimination of several manufacturing steps [4]. CAD/CAM production involves three consecutive steps: scanning, CAD modeling, and CAM production. The scanner is the data acquisition system that records the 3D geometry of the infrastructure and converts the actual dental model into virtual dental model. The CAD component virtually designs the 3D contour of the final implant component. The CAM system produces the actual implant component according to the virtual design. In implant dentistry, the implant abutments and frameworks are produced by milling at a central production facility. Examples of these systems are Procera (Nobel Biocare), Etkon (Straumann), CAMStructure (Biomet 3i), and Atlantis (Astra Tech). 

Custom CAD/CAM abutments combine most of the advantages of stock and cast custom abutments [29]. In addition to a predictable fit and durability, all the prosthesis parameters are modifiable including the emergence profile, thickness, finish line location, and external contour. This is performed by copying resin or wax pattern manufactured by a dental technician or by computer software modelling [29, 30]. Initially, CAD/CAM was used to fabricate implant components from titanium and titanium alloy. To date, CAD/CAM is the only way of producing implant components from high-strength ceramics such as densely sintered alumina and partially stabilized zirconia.

In relation to implant prosthodontics, the use of CAD/CAM has three merits: accuracy (or precision of fit), durability, and simplicity of construction. Each of these points of merit are discussed as follows.

### 4.1. Accuracy

**Figure 1:**
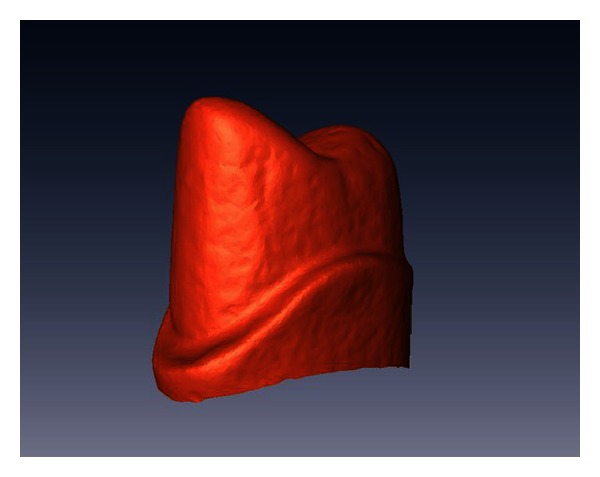
Scanned image of a prepared tooth. The accuracy of the scanning is dependent on the overall smoothness and definition of the preparation.

The assumption that CAD/CAM production is more accurate than the lost wax/casting technique is based on minimal human intervention and bypassing several fabrication steps such as waxing, investing, casting, and polishing. The literature that evaluated the accuracy of tooth-supported CAD/CAM restorations did not confirm that the accuracy of CAD/CAM copings improved when compared with conventionally produced copings [31, 32]. Although the level of fit of CAD/CAM copings was within the acceptable range, a degree of misfit was reported in relation to the restoration margin and the internal fitting surface [31]. This was primarily attributed to the irregularities and variation on the prepared tooth surface that is recorded in the scanned digital image (Figure 1). From an engineering perspective, irregular surfaces are more difficult to scan which results in excessive surface noise. Subsequent image processing and noise elimination can cause rounding of the edges and loss of image sharpness [33, 34]. In relation to CAM design, several authors have proposed mathematical algorithms to compute the restoration external anatomy that fits within the arch and against the opposing dentition [35, 36]. Still, a discrepancy of up to 0.5 mm can be anticipated on the occlusal surface which will require manual adjustment [35]. The CAM process is dependent on the diameter of the smallest bur which is about 1 mm [30]. Restoration features with a smaller diameter might not be accurately produced. To overcome this problem, the CAD/CAM system might excessively mill the workpiece to compensate for the minor features [37].

On the contrary, implant CAD/CAM abutments and frameworks have been reported to be consistently better fitting than conventional cast components. In relation to implant abutments, the vertical gap for titanium and zirconia abutments was in the range of 2.5–3.2 *μ*m [38] which was comparable to stock implant abutments. The difference between milled titanium and zirconia abutments was insignificant [38]. Likewise, the rotational freedom for CAD/CAM abutments was reported to be less than 3° regardless of abutment materials [39]. With implant frameworks, CAD/CAM production has been reported to be at least as accurate as the most accurate implant framework fabrication method and with a tendency to provide the most consistent outcome [28]. This indicates the predictability of obtaining an accurate fit in comparison with other fabrication techniques. The vertical fit of CAD/CAM frameworks ranged from 1 to 27 *μ*m which was significantly better than cast implant frameworks [25, 26]. In addition, a similar level of fit was observed for implant CAD/CAM frameworks produced from zirconia and titanium [40]. In contrast to the conventional casting technique, the level of precision does not seem to be affected by the span of the framework as similar levels were observed for complete and partial arch frameworks [25, 26, 40]. However, more studies are required to confirm this observation.

**Figure 2:**
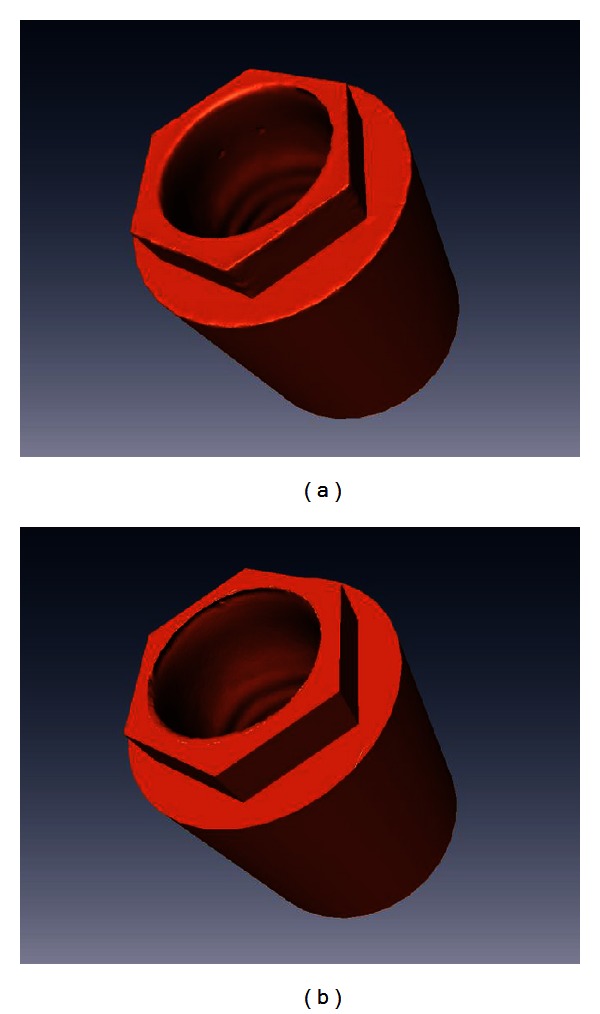
(a) Initial image after scanning implant replica. The accuracy of the final image is influenced by smoothness of the sharp corners. (b) Reverse engineering of implant replica can reproduce exact implant dimensions.

An engineered implant surface is advantageous in being smoothly machined with defined features that facilitates recording the exact geometry with minimal irregularities. In addition, since the implant surface is composed of defined dimensional parameters, the scanned implant surface can be reverse engineered to reproduce precise implant geometry (Figure 2). Consequently, the purpose of the scanner is to register the implant position rather than recording the surface details; eventually, there is less reliance on the acuity of the scanning procedure.

Regarding the external morphology of an implant, as definitive occlusal contacts are not intended to be established on implant abutments or frameworks, the external morphology is more forgiving than fully contoured restorations that are supposed to fit precisely in occlusion. Producing an implant abutment and framework with an external surface clearance is therefore simpler and more predictable than the completed restoration. Although the correct component design and clearance is determined according to the definitive restoration material, a minor discrepancy can be easily rectified in the definitive restoration with manual ceramic veneering. 

**Figure 3:**
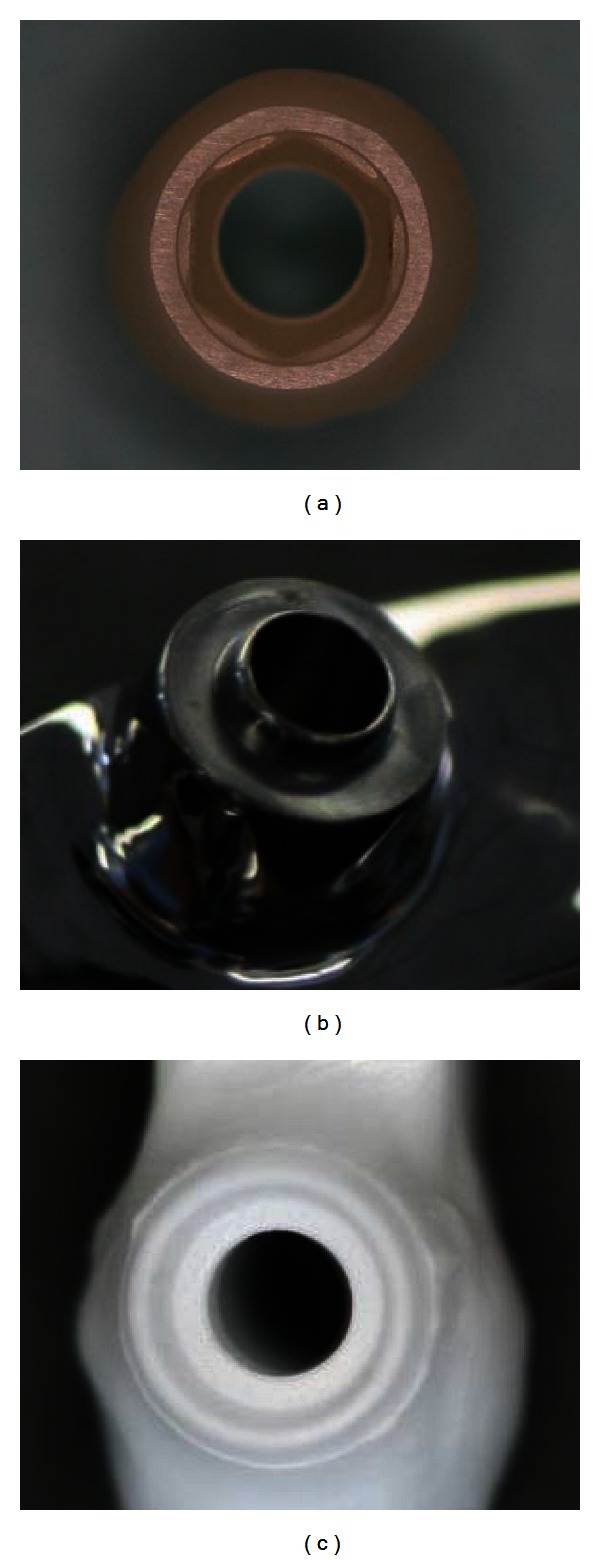
(a) Engaging fitting surface of zirconia CAD/CAM abutment. Non-engaging fitting surfaces of titanium (b) and zirconia (c) frameworks. Accurate nonengaging surfaces can be milled since all their features are larger than the smallest milling bur.

The milling procedure is less likely to cause a fit discrepancy for implant abutments and frameworks. For an implant abutment, the ingots are available with a precisely machined fitting surface [41]. Subsequently, the milling procedure is restricted on the external surfaces without altering the precision of the fitting surface. In contrast, for implant frameworks, the fitting surfaces are produced by milling (Figure 3(a)); however, as non-engaging fitting surfaces are produced, the milling procedure will not encounter sharp edges during production. As a result, because all the fitting surface features have diameters well above the diameter of the smallest milling bur, production of an accurate fitting surface is reliably achievable (Figures 3(b) and 3(c)).

### 4.2. Durability

The durability of CAD/CAM abutments and frameworks can be enhanced by (1) material durability and (2) design customization. The use of an industrial manufacturing process with minimal human intervention is anticipated to control the quality and reduce manufacturing deficiencies. For many years, titanium has been the gold standard due to its mechanical strength and biocompatibility. The advantages of milled titanium abutments and frameworks have been well supported by clinical studies [42, 43].

In an aesthetic-conscious society, there is a demand for an aesthetic and durable implant restoration. This had led to the adoption of high-strength abutments and frameworks for implant prosthesis. Traditionally, the application of ceramic material in prosthodontics has been associated with more frequent mechanical complications. The advent of high-strength ceramics such as alumina and zirconia has enabled researchers to apply these materials to implant prostheses. This has been largely facilitated by the use of the CAD/CAM manufacturing process because it is the only method available to fabricate high-strength ceramics [44]. Because the durability of zirconia is superior to alumina [45, 46], zirconia has attracted much more clinical attention.

With implant abutments, when comparing the fracture resistance of titanium and zirconia abutments in vitro, titanium abutments have been found to be more durable [22, 47]. It was also found, however, that zirconia abutments were durable enough to withstand an applied occlusal load in the range of 300–460 N [22, 48–51]. Since these values were above the maximal physiological occlusal forces on the anterior teeth, zirconia abutments were recommended for use with anterior implant restorations [8, 52], where the physiologic maximal occlusal forces reach approximately 300 N. For the posterior implant restoration, the routine use of zirconia abutments needs to be validated [53, 54].

With implant frameworks, there are currently a limited number of laboratory and clinical studies. Much of the information has been obtained from studies that used zirconia for tooth-supported fixed partial dentures. Under static loading, Kokubo et al. found that 3-unit zirconia frameworks could withstand forces ranging from 475 to 722 N [55]. The limited clinical studies have shown that zirconia frameworks are relatively stable with the complications occurring with the veneering ceramic [52]. The complications at the framework level [56] and veneering ceramic level [57] have been found to increase as the span of the prosthesis increases.

Zirconia abutments and frameworks benefit from full customization as this will ensure minimal zirconia adjustment and maximal material bulk for durability. Kohal et al. found that modifying the zirconia stock abutments with a diamond bur caused a decrease in the fracture strength [58]. Since CAD/CAM produces zirconia workpieces that require no subsequent alteration, unnecessary weakening is avoided. Maximal abutment and framework thickness is desirable and increases the fracture resistance. Following retrieval of fractured zirconia abutments, Aboushelib and Salameh observed that 2 out of 5 abutments fractured due to overreduction of the axial walls [6]. Further, Nguyen et al. reported that wider CAD/CAM abutments are less likely to fracture than narrower abutments [59], and Ohlmann et al. found that thickened zirconia frameworks exhibited higher fracture resistance [60]. 

CAD/CAM production will also facilitate the durability of the veneering ceramic by contouring the zirconia abutment according to the morphology of the definitive crown [29]. Such anatomical contouring aims to reduce the thickness of the veneering ceramics and has been found to reduce the risk and severity of ceramic chipping [9–11]. 

To date, the limited clinical studies have revealed a comparable outcome for zirconia and titanium abutments [61, 62]; however, more data is required regarding the clinical performance of zirconia frameworks prior to the routine recommendation of zirconia prostheses. Unfortunately, clinical studies on the performance of partial- and complete-arch fixed zirconia prostheses [63, 64] have revealed that the percentage of veneering ceramic failure is very high, ranging from 50% to 90% [63, 64]. As a result, the authors have recommended caution prior to widespread use of zirconia for partial- and complete-arch prostheses [64]. 

The risk of veneering ceramic fracture is expected to be minimized in the future by the continuously improving veneering strategies. Methods like heat-pressing the veneering ceramic [65, 66] or slow cooling of the veneered zirconia restoration [67] are showing an encouraging outcome. On the other hand, monolithic zirconia restorations, where the implant restoration is milled to the final contour without subsequent ceramic veneering, have had an encouraging outcome in early case reports [68, 69].

The development of CAD/CAM has occurred in parallel with material science advancement. It is therefore very likely that different aesthetic materials, such as polymer-infiltrated ceramics, will be used in the fabrication of CAD/CAM restorations [70, 71]. These materials will overcome some inherent problems of ceramics such as brittleness, risk of chipping, and difficulties with reparability.

### 4.3. Simplified Protocol

In comparison to the lost wax/casting protocol, CAD/CAM is much simpler and requires less technical time and involvement. This applies to the fabrication of the implant abutment and framework. The steps eliminated by CAD/CAM require more materials manipulation and precise operator handling. Instead of waxing and casting, the whole CAD/CAM process is fully automated following the scanning step. A well-designed implant CAD/CAM abutment or framework rarely requires additional intervention by the dental technician. Subsequently, the predictability of the final result of CAD/CAM will reduce the clinical time involved in evaluating the component quality. Because of this, some operators have proposed omitting the framework try-in step [25]. 

A recently introduced CAD/CAM abutment system is the Encode abutment (Encode; Biomet 3i, Palm Beach Gardens, Fla). This system involves the utilization of a coded healing abutment that indicates the implant depth, diameter, hex orientation, location of gingival tissues, and orientation of the implant. Following a closed tray impression and master model fabrication, the model is scanned and the exact implant location is determined virtually. Subsequently, the information from the Encode abutment is used to mill a titanium or zirconia abutment. In addition, an implant analogue is fitted on the master model with the aid of a robotic system. Following cast alteration, the CAD/CAM abutment is fitted on the implant analogue and sent to the lab for definitive crown fabrication. This system has the advantages of simplicity, overcoming the open tray impression procedure, and reducing the clinic time required to take an impression [2, 72–74]. This concept is therefore more likely to be preferred by the patient. It is speculated from clinical reports that the tissue response will be more favourable as fewer interventions are necessary, reducing the risk of tissue irritation. The manufacturer claims that correction of up to 30° implant angulation is also possible [2, 73]. Further simplification was envisioned using digital intraoral scanning instead of the laboratory scanning which will omit clinical impression procedure [74, 75]. This system however is only applicable to 3i implants. In addition, clinical customization of the soft tissue profile is limited [72].

Following the comparison of the Encode abutment impression technique and open tray implant level impression technique, Eliasson and Örtorp found that both of the techniques had minimal 3D discrepancies and rotational errors, although the open tray technique was more accurate [75]. Since the Encode system is used to fabricate individual single implant abutments, minimal orientation errors will not compromise the implant-abutment junction. Further, because the final restoration is cement-retained, any rotational errors can be compensated by the intermediate cement layer [76].

More recently, chair-side construction of an implant abutment can also be achieved using CAD/CAM and is available using the Cerec system (Sirona). This concept involves intraoral scanning of a prefabricated titanium cylinder followed by designing and milling a definitive zirconia abutment to the optimal contour. The zirconia abutment is adhesively bonded on the prefabricated titanium cylinder [77]. Other authors have discussed the use of a similar protocol to construct a provisional implant crown [78]. The main advantage of this system is the omission of the impression step as well as ensuring the accuracy of the prefabricated abutments.

## 5. Conclusion

It is indisputable that the CAD/CAM application can be used to facilitate the restoration of oral implants. The machined and evenly designed implant surface enhances the CAD/CAM performance. Precision of fit, durability, simplicity, and aesthetic material application are the main advantages of CAD/CAM in implant dentistry; however, more clinical studies are required to validate the superiority of CAD/CAM restorations. The advantages of CAD/CAM will most likely lead to an exponential growth in the utilization of this technology in implant dentistry.
